# microRNAs in nociceptive circuits as predictors of future clinical applications

**DOI:** 10.3389/fnmol.2013.00033

**Published:** 2013-10-17

**Authors:** Michaela Kress, Alexander Hüttenhofer, Marc Landry, Rohini Kuner, Alexandre Favereaux, David Greenberg, Josef Bednarik, Paul Heppenstall, Florian Kronenberg, Marzia Malcangio, Heike Rittner, Nurcan üçeyler, Zlatko Trajanoski, Peter Mouritzen, Frank Birklein, Claudia Sommer, Hermona Soreq

**Affiliations:** ^1^Department of Physiology and Medical Physics, Division of Physiology, Medical University InnsbruckInnsbruck, Austria; ^2^Medical University InnsbruckInnsbruck, Austria; ^3^UMR 5297, Interdisciplinary Institute for Neuroscience, Centre National de la Recherche Scientifique, University of BordeauxBordeaux, France; ^4^University HeidelbergHeidelberg, Germany; ^5^Hebrew University of JerusalemJerusalem, Israel; ^6^University Hospital BrnoBrno, Czech Republic; ^7^The European Molecular Biology LaboratoryMonterotondo, Italy; ^8^King’s College LondonLondon, UK; ^9^University Hospital WürzburgWürzburg Germany; ^10^Exiqon A/SVedbaek, Denmark; ^11^University Hospital MainzMainz, Germany; ^12^Laboratory of Molecular Neuroscience, Department of Biological chemistry, Hebrew University of JerusalemJerusalem, Israel

**Keywords:** chronic pain, biomarker, polymorphism, miRNA-based diagnostics, miRNA expression patterns, miRNA polymorphisms, antagomir, miRNA-based analgesic

## Abstract

Neuro-immune alterations in the peripheral and central nervous system play a role in the pathophysiology of chronic pain, and non-coding RNAs – and microRNAs (miRNAs) in particular – regulate both immune and neuronal processes. Specifically, miRNAs control macromolecular complexes in neurons, glia and immune cells and regulate signals used for neuro-immune communication in the pain pathway. Therefore, miRNAs may be hypothesized as critically important master switches modulating chronic pain. In particular, understanding the concerted function of miRNA in the regulation of nociception and endogenous analgesia and defining the importance of miRNAs in the circuitries and cognitive, emotional and behavioral components involved in pain is expected to shed new light on the enigmatic pathophysiology of neuropathic pain, migraine and complex regional pain syndrome. Specific miRNAs may evolve as new druggable molecular targets for pain prevention and relief. Furthermore, predisposing miRNA expression patterns and inter-individual variations and polymorphisms in miRNAs and/or their binding sites may serve as biomarkers for pain and help to predict individual risks for certain types of pain and responsiveness to analgesic drugs. miRNA-based diagnostics are expected to develop into hands-on tools that allow better patient stratification, improved mechanism-based treatment, and targeted prevention strategies for high risk individuals.

## INTRODUCTION

Human chronic pain disorders are bio-psycho-social diseases, which are difficult to treat due to their diversity. Chronic pain syndromes that develop after nerve damage, trauma or surgery are characterized by persistent and severe pain; they induce anxiety and depression and greatly impair patients’ quality of life. One out of five Europeans suffers from chronic pain with most reporting that they endure it for more than two years (Breivik et al., 2006; Baker et al., 2010). Due to direct and follow-up costs they constitute a heavy burden for the health system (Phillips, 2006).

Of the painful neuropathies, the most frequent, painful diabetic polyneuropathy is a common complication of diabetes mellitus occurring in up to 20% of patients (Sommer, 2003; Sadosky et al., 2008). Good glycemic control can reduce the incidence of diabetic polyneuropathy but not painful diabetic polyneuropathy (PDPN) for which only symptomatic therapy of low to moderate efficacy is available to date (Vincent et al., 2011). Cellular mechanisms are emerging that include the classical changes of the diabetic milieu (Bierhaus and Nawroth, 2012; Bierhaus et al., 2012) however various studies have also identified signatures of neuroinflammation as critical components of painful diabetic polyneuropathy (Pabreja et al., 2011; Vincent et al., 2011). Pathological neuro-immune communication has also been associated with painful neuropathy that occurs in up to 50% of patients with traumatic peripheral nerve injury as a consequence of accidents, warfare or surgical procedures (Myers et al., 2006; Ciaramitaro et al., 2010; Birch et al., 2012). Also the neurogenic complex regional pain syndrome (CRPS) occurring as a complication of bone fracture, tissue injury or surgical interventions has a neuro-inflammatory component (Parkitny et al., 2013). In the majority of cases symptoms grossly resolve, however in 30% of patients pain symptoms persist or even intensify (Marinus et al., 2011). The beneficial effect of therapy with glucocorticosteroids in the acute phase of CRPS supports pathophysiological mechanisms associated with neuro-immune dysfunction (Üceyler et al., 2007a; Fischer et al., 2010; Marinus et al., 2011). Thus, converging evidence suggests that neuro-immune alterations in the peripheral and central nervous system play a major role in the general pathophysiology of neurogenic and neuropathic pain (McMahon and Malcangio, 2009; Kuner, 2010). Non-coding RNAs (ncRNAs), including microRNAs (miRNAs) and Piwi-binding piRNAs, are intimately associated with normal cellular as well as pathological processes (Mattick, 2004; Hüttenhofer et al., 2005; Hüttenhofer and Schattner, 2006). In this review we will focus on miRNAs since they are most extensively studied so far.

Various diseases, including neuropathic pain disorders, reveal unique miRNA expression signatures that can be exploited as diagnostic and prognostic markers. Recent reports on miRNA modulation of both neuronal and immune processes further predict therapeutic potential for manipulating disease-modified miRNAs in diseases affecting both the immune system and brain function, such as neuropathic pain disorders, Alzheimer’s disease, Parkinson’s disease, multiple sclerosis, and anxiety-related disorders (Soreq and Wolf, 2011; O’Connor et al., 2012).

**FIGURE 1:**
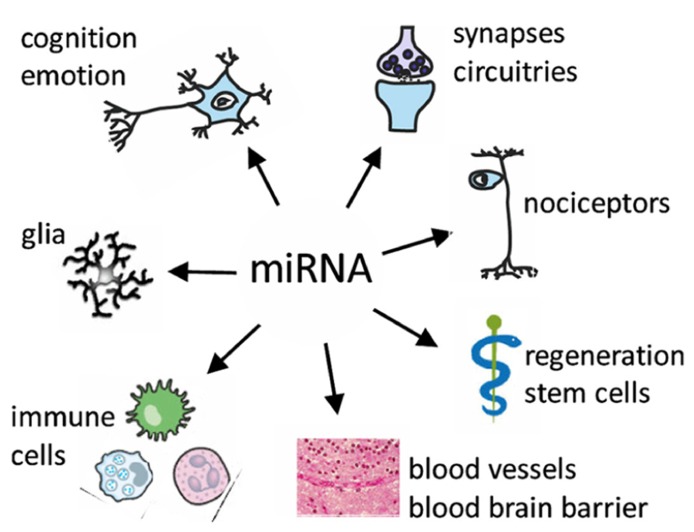
**Targets of miRNA regulation networks**.

miRNAs that function within both the nervous and the immune systems possibly act as “negotiators” between these two interacting compartments (**Figure 1**). These “neurimmiRs” primarily target transcription factor genes or other regulatory genes, which enables simultaneous modulation of both immune and neuronal processes including cognition through direct or indirect alterations of neuron–glia or brain-to-body signaling (Soreq and Wolf, 2011). Thus, a given miRNA controls multiple cellular pathways, and miRNAs can act as “master switches” of the transcriptome or proteome, regulating multiple gene products and orchestrating multiple pathways including genes that encode cellular enzymes, trophic factors, receptor proteins, and ion channels many of which are individually pursued as drug targets.

Pain conditions have been suggested to deregulate the expression of miRNAs in pain pathways from primary afferent nociceptors to brain areas associated with emotional components of pain perception (Bai et al., 2007; Aldrich et al., 2009; Kusuda et al., 2011; Imai et al., 2011; Poh et al., 2011; von Schack et al., 2011). miRNAs are frequently deregulated and expressed at aberrant levels in diseased tissue, and first evidence suggests that this applies to neurogenic pain in CRPS (Orlova et al., 2011). Altered miRNA expression is frequently a consequence of genetic mutations, which may also cause loss or gain of function (Mishra and Bertino, 2009). This may account for inter-individual variation of pain sensitivity. However, the functional consequences of polymorphisms in miRNA genes and/or their binding sites, the downstream targets of miRNAs and the mechanisms by which miRNAs regulate circuitries and processes modulating nociception and endogenous analgesia are as yet unresolved.

Therapeutic miRNA regulation has been thoroughly studied and widely established in cancer research but its impact and the therapeutic prospects of miRNAs in the pain field are largely unexplored. Manipulation of miRNAs offers the possibility to control multiple targets including neuro-immune interactions, nociceptive processing and cognitive pathways. Both miRNAs and their isomiRNA versions are likely to each interact with many different targets, which may lead to downstream changes either due to the direct suppression of these targets or because of regulatory effects of those targets. Such downstream effects may be rather elaborate and are defined by some researchers “off-target” effects. However, we find that this definition may be misleading as it assumes that the physiological role of each miRNA is limited to the suppression of its direct targets. It is expected that miRNAs and miRNA derivatives will have few, if any, sequence-specific “off-target” effects. Thus, miRNA based diagnostics and therapeutics may have superior advantages by targeting multiple pain-associated genes and miRNA-based drugs may be the most appropriate therapy for the prevention or treatment of neuropathic pain.

## BIOMARKERS FOR NEUROPATHIC AND NEUROGENIC PAIN SYNDROMES

Painful diabetic polyneuropathy is the most frequent painful neuropathy occurring in up to 20% of diabetic patients (Sommer, 2003; Sadosky et al., 2008). CRPS is an extremely painful condition that occurs in some patients after bone or tissue injury and peripheral nerve injury (traumatic neuropathy) and results in chronic neuropathic pain in many of these patients. These well-characterized albeit aetiologically diverse (metabolic, inflammatory, traumatic) neuropathic/neurogenic pain syndromes cover a spectrum of mechanisms underlying chronic pain. Nevertheless, the medical need for these syndromes is prevalent, and each of them is prototypic for an entire group of pain disorders.

It is unclear why diabetic neuropathy or traumatic neuropathy are painful in some instances and painless in others or why some patients develop CRPS after bone fracture, and why some recover from CRPS and others do not (Marinus et al., 2011). Thus, as yet unknown factors determine whether a given disorder entails chronic neuropathic pain. A first approach to be able to predict the individual risk of pain chronification was to use sensory phenotypes as surrogate markers for possible underlying mechanisms. Quantitative sensory testing (QST) is now well established but is still insufficient to disentangle specific pathophysiological mechanisms of chronic pain (Baron et al., 2012). One of the major hindrances in translating such findings into better therapy of neuropathic and neurogenic pain syndromes is the complexity of their pathophysiology. It is well known that alterations in many processes including ion channels, inflammatory mediators, neurotrophic factors, synaptic plasticity, and de- and regeneration, are involved, and that they even change during the course of the disease (Hehn et al., 2012). Therefore, a search for better and more specific diagnostic trait and state markers is one of the prerequisites for successful treatment in the future. Circulating miRNAs are detectable in body fluids including blood and cerebrospinal fluid and may be useful as novel biomarkers amenable to clinical diagnostic applications for various types of disease (Cogswell et al., 2008; Orlova et al., 2011; Ajit, 2012; Weiland et al., 2012; Machida et al., 2013). Therefore, it should be likewise promising to carefully assess which circulating miRNAs and novel ncRNAs are associated with neurogenic and neuropathic pain syndromes and may emerge as reliable diagnostic biomarkers for painful diabetic polyneuropathy, nerve injury pain, CRPS, headache and migraine.

## NEW DRUGGABLE MOLECULAR TARGETS FOR PAIN TREATMENT

Treatment of painful diabetic polyneuropathy is far from satisfactory in many patients although this is the most intensely studied painful neuropathy in randomized controlled trials (RCTs). National and international guidelines differ in their recommendations about first and second line treatment choices. While pregabalin is favored by some (Bril et al., 2013), duloxetine or even tricyclic antidepressants are first choice in others (NICE-guideline; Attal et al., 2010; Dworkin et al., 2007). All of these drugs have adverse effects on diabetes. Furthermore, mean treatment effects comprise only two points of pain reduction on a 11-point Likert scale. In other types of neuropathy, like traumatic neuropathy or the frequent inflammatory types, there is little or no data at all from RCTs on pain treatment. Even worse, treatment of CRPS is neither standardized, nor satisfactory, nor based on multicentre RCTs. From single center studies with very limited patient numbers some evidence exists for anti-inflammatory treatment by corticosteroids or bisphosphonates in acute but not chronic stages, and for behavioral therapy for selected patients in chronic stages (de Tran et al., 2010). For the most frequently used invasive treatment modalities such as sympathetic blockers no RCT evidence of efficacy is available (Straube et al., 2010). Thus, more efficacious and specific medications are needed for both neurogenic and neuropathic pain syndromes.

**FIGURE 2:**
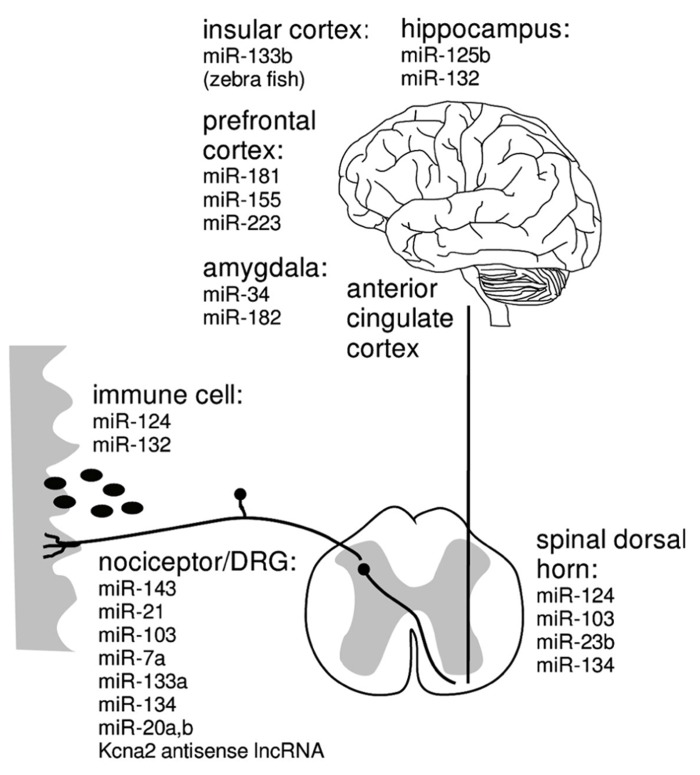
**miRNA that may be causally associated with maintained neuropathic pain in immune cells (Shaked et al., 2009; Soreq and Wolf, 2011; Ponomarev et al., 2013), nociceptors/DRG (Ni et al., 2012; Sakai and Suzuki, 2013), spinal cord (Brandenburger et al., 2012; Favereaux et al., 2011; Im et al., 2012; Ni et al., 2012; Willemen et al., 2012), insular cortex (Sanchez-Simon et al., 2010), amygdala (Meerson et al., 2010; Haramati et al., 2011; Griggs et al., 2013), prefrontal cortex (Poh et al., 2011), hippocampus (Edbauer et al., 2010)**.

Both, the novel and specific mode of action and the ability to function as master switches of entire signaling networks has triggered enthusiasm for miRNAs as promising therapeutic targets although relatively little is known about the mechanisms of cellular uptake, storage and mode of action of miRNA modulators (van Rooji and Olson, 2012). In several rodent pain models, deregulated expression of miRNAs was found in pain pathways from primary afferent nociceptors to brain areas associated with emotional components of pain perception (**Figure 2**; Bai et al., 2007; Aldrich et al., 2009; Kusuda et al., 2011; Imai et al., 2011; Poh et al., 2011; von Schack et al., 2011). First evidence supporting a future for analgesic miRNA treatment comes from mice intrathecally receiving miR-124, miR-103 or miR-23b which are reported to prevent and treat persistent inflammatory and neuropathic pain (Favereaux et al., 2011; Imai et al., 2011; Willemen et al., 2012). Despite the fact that these miRNA treatments reduced signatures of synaptic modification, neuroinflammation and microglial response, the full extent and the mechanisms of the analgesic effect are not understood to date (Favereaux et al., 2011; Willemen et al., 2012).

## CIRCUITRIES AND PROCESSES MODULATING NOCICEPTION AND ENDOGENOUS ANALGESIA

Various studies have identified signatures of neuroinflammation as critical components of diabetic polyneuropathy (Pabreja et al., 2011; Vincent et al., 2011) in addition to the cellular mechanisms that include the classical changes of the diabetic milieu (Bierhaus et al., 2012; Bierhaus and Nawroth, 2012). Pathological neuro-immune communication has been associated with painful neuropathy following traumatic peripheral nerve injury (Myers et al., 2006; Ciaramitaro et al., 2010; Birch et al., 2012). Moreover, CRPS occurring as a complication of bone fracture or tissue injury results from neurogenic inflammatory processes (Goebel, 2011). In humans, a systemic pro-inflammatory profile distinguishes painful from painless neuropathy, and a local pro-inflammatory profile is part of the pathophysiology of small fiber neuropathy (Üceyler et al., 2007b, 2010). Specialized peripheral neurons, the nociceptors sense inflammatory or neuropathic conditions and respond with increased excitability and sensitivity leading to persisting pain and hyperalgesia (Costigan and Woolf, 2000; Sommer and Kress, 2004; Berta et al., 2008; Üceyler et al., 2009). However, mice lacking receptors for pro-inflammatory mediators in their nociceptor neurons are frequently protected from certain signatures of pathological pain (Andratsch et al., 2009; Schweizerhof et al., 2009; Mair et al., 2011; Quarta et al., 2011). The deficiency in anti-inflammatory cytokines in patients with CRPS (Üceyler et al., 2007a) together with beneficial effect of therapy with glucocorticosteroids support pathophysiological mechanisms associated with neuro-immune dysfunction (Fischer et al., 2010).

Inflammatory processes are also activated in the spinal cord upon peripheral nerve injury and involve microglia activation and leakage at the blood nerve barrier along the entire neuraxis (McMahon and Malcangio, 2009; Beggs et al., 2010, 2012). Microglia activation occurs in diabetic neuropathy in rodents (Wodarski et al., 2009; Beggs et al., 2012; Talbot and Couture, 2012) and has been recognized to be critical for the maintenance of neuropathic pain via the release of pro-nociceptive mediators (Clark et al., 2007). Leakage of the blood nerve barrier or the blood spinal barrier is just emerging in the pathophysiology of neuropathic pain accompanied by changes in tight junction proteins (Echeverry et al., 2011). Tight junction proteins which are critically involved in maintaining the blood–brain barrier like claudin-1 are also new targets, e.g., of miR-155 (Qin et al., 2013).

Deregulated miRNAs can be a consequence or cause of local inflammatory processes such as regulation of nociceptor sensitisation by controlling phospholipase A2 activation (Sun et al., 2012). Analyses of expression profiles of dorsal root ganglia (DRG) containing nociceptor cell bodies reveal that particular miRNAs are deregulated in rodent pain models giving rise to deregulation of miRNA-targeted ion channel expression patterns and metabotropic receptor transcripts in peripheral neurons which presumably cause nociceptor dysfunction (Zhao et al., 2010; von Schack et al., 2011). miRNAs are universal regulators of differentiation, activation and polarization of microglia in normal and inflammatory conditions (Ponomarev et al., 2013). Microglia and macrophage activity is suppressed by specific miRNAs, e.g., miR-124, and it is therefore anticipated that miRNA regulation is critically involved in endogenous inhibition and resolution of inflammation by e.g., resolvins (Ponomarev et al., 2011; Recchiuti et al., 2011). Certain miRNAs are substantially suppressed in glucocorticoid-treated thymocytes by reduced expression of the key miRNA processing enzymes Dicer, Drosha, and DGBR8/Pasha (Smith et al., 2010). This observation is of great relevance since CRPS for example is regarded a prototype disorder of failed termination of inflammation (Birklein and Kingery, 2009). The spinal release of immune modulators affects both spinal synaptic processes and local inhibitory circuits, possibly by classical cytokine-prostaglandin signaling and dys-inhibition of e.g., glycinergic spinal control (Samad et al., 2001; Harvey et al., 2004). Plastic changes at synapses in the spinal dorsal horn promote neuropathic and neurogenic pain via mechanisms involving enhanced nociceptive transmission but also inhibition of spinal endogenous analgesic circuits (Hartmann et al., 2004; Harvey et al., 2004; Fossat et al., 2007; Sandkühler, 2007, 2009; Pernïa-Andrade et al., 2009; Zeilhofer et al., 2009; Fossat et al., 2010; Laffray et al., 2012).

For a few miRNAs and long ncRNAs, downstream target proteins have been reported. For example, a conserved long ncRNA seems to modulate sensory neuron excitability by activation of a transcription factor MZF and downregulation of *Kcna2* potassium channel expression and this has been causally associated with neuropathic pain (Zhao et al., 2013). In addition, the functional consequences of miR-103 regulation of voltage-gated Cav1.2 calcium channels and intrinsic excitability of spinal projection neurons have been demonstrated (Favereaux et al., 2011). It is well accepted that certain hereditary forms of migraine are associated with polymorphisms of voltage-gated calcium channels Cav2.1 and Cav2.2 (Pietrobon and Striessnig, 2003). Novel evidence suggests that in particular endogenous pain control systems including GABAergic and opioidergic synaptic signals are down-regulated by miRNAs such as miR-134 or miR-181a (Ni et al., 2012; Sengupta et al., 2013). Some of them link miRNAs like let-7 or miR-339 to opioid tolerance (He et al., 2010; He and Wang, 2012; Wu et al., 2013). In analogy, miRNA neuronal dys-regulation should not only apply to neurogenic or neuropathic pain but very likely the same principles and pathways should apply to other pain syndromes like headaches and in particular hereditary and other forms of migraine.

## COGNITIVE, EMOTIONAL AND BEHAVIORAL COMPONENTS OF PAIN

Neuropsychological alterations are present in 65 % of CRPS patients and in particular cognitive impairment and deficits of emotional decision-making may impact their quality of life especially in risky, emotional situations (Apkarian et al., 2004). Emotional deficits and functional alterations in corresponding brain regions are reported in chronic CRPS patients and pain-related fear is one of the strongest predictors of disability in chronic pain disorders (Geha et al., 2008; de Jong et al., 2011).

Specific areas in the brain are actively involved in pain perception and behavior in humans and rodents and structural brain changes are associated with sensory and emotional function in rodent long-term neuropathic pain. In particular, decreased volumes of primary somatosensory and frontal cortex, retrosplenial and entorhinal cortex, anterior cingulate cortex and insula are maintained for months (Seminowicz et al., 2009). Specifically, abnormalities in hippocampus volume are observed in human CRPS and the mouse spared nerve injury (SNI) model. Similar to CRPS patients, SNI mice show increased anxiety like behavior and abnormal contextual fear extinction and this is associated with reduced extracellular signal-regulated kinase (ERK) expression, decreased neurogenesis and altered synaptic plasticity (Kodama et al., 2007; Mutso et al., 2012). Mice with experimental neuropathic pain also show cognitive deficits in novel object recognition and this is associated with deregulation of glycinergic neurotransmission in the hippocampus (Kodama et al., 2011), and may relate to reported enhanced quantal neurotransmitter release in the anterior cingulate cortex of mice with neuropathic pain (Toyoda et al., 2009). Dopaminergic and glutamatergic inputs from amygdala, hippocampus and prefrontal cortex to the nucleus accumbens participate in the putative emotional control circuits and recent human brain activity studies have examined the nucleus accumbens in the emotional aspects of pain processing (Baliki et al., 2010). These reports further link chronic pain with emotional dysfunction, and maladaptive responses of the nucleus accumbens in neuropathic pain have recently been associated with deregulated miRNAs in this region (Imai et al., 2011).

Brain-specific miRNAs are emerging as regulators of cognition, neuronal plasticity and memory by manipulating synapse structure and function, and specific miRNAs not only control cognition and emotional processes but also neuro-immune communication in the brain (Bredy et al., 2011; Soreq and Wolf, 2011). Mental retardation has been associated with miR-125b, miR-132 and other miRNAs and this arises from effects on dendritic spine morphology and synaptic physiology at hippocampal neurons. AMPA-mediated miniature mEPSC amplitude and frequency are reduced by neuronal over-expression of miR-125b and increased by miR-132 and this is due to differential regulation of glutamate NR2A and NR2B receptor mRNA levels (Edbauer et al., 2010). Other glutamate receptor subunits in the brain are regulated by dopamine through miR-181a which has recently been associated with the pain system (Saba et al., 2012). miR-132 is a highly interesting brain specific miRNA since it is up-regulated by brain derived neurotrophic factor (BDNF) and other growth factors in cortical neurons and this results in an increased expression of synaptic proteins including glutamate receptors (NR2A, NR2B and GluA1), an effect that is attenuated by glucocorticoids (Kawashima et al., 2010; Numakawa et al., 2011). Hippocampal miR-132 mediates stress-inducible cognitive deficits through acetylcholinesterase as a downstream target and specifically in the amygdala miR-34 is associated with the repression of stress-induced anxiety (Haramati et al., 2011; Shaltiel et al., 2013). More generally, happiness, anxiety and depression seem to depend on miRNA expression levels. Specific miRNAs are deregulated in patients suffering from depression and anxiety, and in pre-clinical models of psychological stress (Meerson et al., 2010). Moreover, psychoactive agents, including antidepressants and mood stabilizers, utilize miRNAs as downstream effectors (O’Connor et al., 2012). This further links neuropathic pain to emotional disorders and to the clinical benefit of antidepressants for pain treatment (Dworkin et al., 2007).

## PAIN PREDISPOSING GENETIC POLYMORPHISMS

There is evidence that chronic pain, pain sensitivity and responsiveness to analgesic opioids show a sufficient heritability to make these phenotypes highly interesting sources for genetic variability which has an influence on pain (Angst et al., 2012; Hocking et al., 2012; Nielsen et al., 2012). Altered miRNA expression is frequently a consequence of genetic mutations, which may also cause loss or gain of function (Mishra and Bertino, 2009). This may account for significant inter-individual variation in the response to painful stimuli and analgesic drugs. Polymorphisms of specific molecular targets may be associated with certain pain phenotypes and this has emerged for example for a specific calcium channel subunit in a *Drosophila* screen that is conserved in mice and humans (Mogil, 2012; Neely et al., 2010). Several meta-analyses are available of the genetics of pain and associated specific loss- or gain of function polymorphisms with altered pain perception (LaCroix-Fralish et al., 2011; Mogil, 2012). A recent genome-wide association (GWA) study revealed three susceptibility loci for common migraine in the general population, however, systematic association studies are unavailable for DPN and CRPS to date (Chasman et al., 2011). In general, genetic studies have helped to understand the role and downstream mechanisms of individual proteins in pain processing, but specific single nucleotide polymorphism (SNP) related pain disorders apply to small numbers of individuals only and so far do not explain the large variability regarding susceptibility to distinct pain disorders or the responsiveness to different pain therapies in the general population (Dworkin et al., 2007; Attal et al., 2010).

The functional consequences of polymorphisms in miRNA genes and/or their binding sites, the downstream targets of miRNAs and the mechanisms by which miRNAs regulate circuitries and processes modulating nociception and endogenous analgesia are entirely unaddressed. SNPs in miRNAs or their target sites are not only bioinformatically predicted to be associated with the pathogenesis of diseases but are also experimentally validated (Wu et al., 2008; Coassin et al., 2010). It is known that SNPs are less common in miRNAs or their target sites than in other parts of the genome which points to the importance of miRNAs for cellular processes. However, on the other hand SNPs in these sites can affect the expression of a large number of genes when the production of the miRNA is influenced by that particular SNP. Moreover, SNPs in target sites of miRNAs can either modulate/disrupt existing binding sites or create new binding sites for the miRNAs that may then influence gene expression. SNPs in these regions have become a major focus of research and some of them are expected to explain pathogenetic mechanisms in disease development (Glinsky, 2008; Haas et al., 2012). For example, miRNA expression is markedly different between normal tissues and tumor tissues although otherwise miRNA expression is strictly controlled. This might be explained by somatic mutations that are introduced during carcinogenesis. The investigation of genetic variants at miRNAs or their target sites and their association with various diseases is only in its infancy. Initial studies show that these RNA chains might also be involved in neurological diseases such as Parkinson’s disease (Martins et al., 2011), Alzheimer’s disease (Serpente et al., 2011) or frontotemporal lobar degeneration (Villa et al., 2011). The identification of SNPs in miRNA related regions of the genome might be advantageous over classical GWA study since individual ncRNAs may control and regulate whole networks and pathways involving a multitude of functional proteins. This may open a new avenue that may potentially improve our understanding of extensive inter-individual differences in patients.

## TRANSLATION OF PRE-CLINICAL AND CLINICAL RESULTS INTO SOLUTIONS FOR THE BENEFIT OF PATIENTS

As stated above, one of the major hindrances in the way of translating such findings into better therapy of neuropathic and neurogenic pain syndromes is the complexity of their pathophysiology, which even changes during the course of disease. Based on and in analogy to recent developments in the oncology field, an improved understanding of the role of miRNAs in neuropathic pain might be highly useful for diagnostic and prognostic assessments. For example, aberrant expression or functional deregulation of miRNAs has been associated with the risk for and progression of malignancies and this knowledge is expected to advance the management of certain cancer types through the development of novel personalized miRNA-based diagnostics and therapies (Dreussi et al., 2012; Rossi and Calin, 2013). Increasing evidence indicates that certain miRNAs may be aberrantly expressed or deregulated in certain individuals after tissue injury or with diabetes. This may be associated with increased risk of pain chronification or even responsiveness to analgesic drugs (Ivanov et al., 2012). Therefore, miRNAs are expected to have potential for personalized pain medicine as biomarkers for risk assessment, drug selection and novel therapies.

Therapeutic miRNA regulation has been thoroughly studied and begins to be established in different types of cancer, and the first miRNA targeted drug has entered phase II clinical trials (Lindow and Kauppinen, 2012). In contrast, the potential therapeutic impact of miRNAs in the pain field is as yet largely unexplored. To date, therapeutic approaches have been restricted to rodent models and intrathecal administration and some inconsistencies have emerged; thus miRNA increases in a disease may be either a cause or a feedback reaction to the observed symptoms. For example, although miR-124 is up-regulated after chronic constrictive nerve injury (CCI), intrathecal administration of miR-124 can prevent and treat persistent inflammatory and neuropathic pain (Willemen et al., 2012). Likewise, miR-132 levels are increased in colon biopsies from patients with intestinal bowel disease which should predictably limit inflammation (Maharshak et al., 2013). Importantly, manipulation of miRNAs offers the possibility to control multiple targets including neuro-immune interactions, nociceptive processing and cognitive and affective pathways. Thus, miRNA based therapeutics may have superior advantages by targeting multiple pain-associated genes and miRNA-based drugs may be the most appropriate therapy for the prevention or treatment of neuropathic and neurogenic pain. At least, recent developments provide an optimistic perspective on the evolution of therapeutic ncRNAs despite the drawback of unresolved obstacles for successful delivery and unknown, however unlikely, off-target effects (Cho, 2012).

**FIGURE 3:**
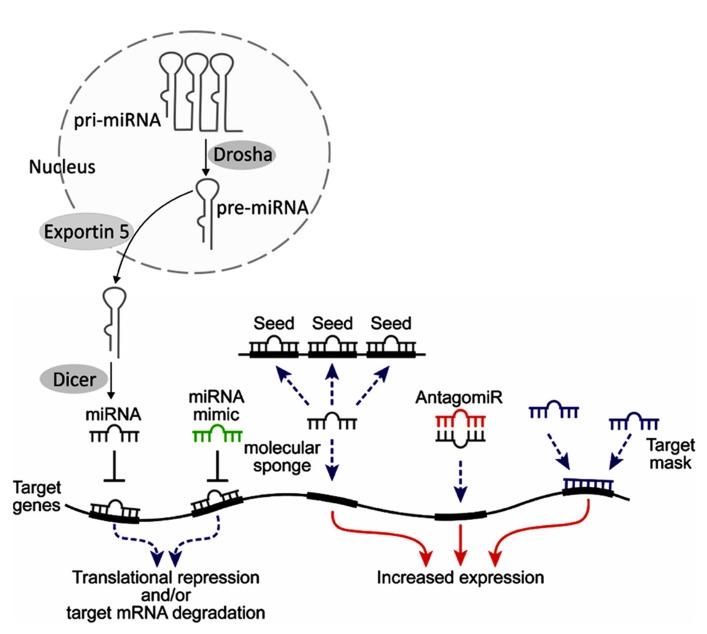
**Endogenous miRNAs are generated from primary (pri-) miRNAs via cleavage by the RNAse Drosha into pre-miRNAs in the nucleus.** They are exported into the cytosol by Exportin 5 and there are cleaved into active miRNAs by the RNAse Dicer. Depending on the degree of homology, miRNAs trigger translational repression or degradation of target mRNAs (for review see He and Hannon, 2004; Bartel, 2009). Therapeutic manipulations of miRNAs may involve various methods. Host tissue miRNAs (gray) bind to complementary sequences, which are often located in the 3′-untranslated region (3′-UTR) of the target genes. This leads to translational repression, often accompanied by degradation. Mimicking this process, miRNA mimics (green) with similar sequences to those of miRNAs may be designed to target the same mRNAs. Such mimics are synthetic oligonucleotides that are chemically protected against nucleolytic degradation. Alternative routes include molecular “sponges,” with several binding sites of a certain miRNA; antagomiRs (red), complementary oligonucleotides to the host miRNA which bind to it and limit its function, and target masks, which bind to part of target miRNAs and compete with their function. Thus, tools exist both for inducing gain of function (red arrows) or loss of function (dashed blue arrows).

Manipulating miRNAs as a therapeutic tool presents significant theoretical and practical challenges that must be overcome before this approach becomes a reality. Specific examples involve two of the more straightforward approaches for miRNA modulation, miRNA mimics and antagomirs (**Figure 3**). miRNA mimics consist of over-expressing specific miRNAs that are reduced in the disease state. This mimic approach could be done by introducing synthetic oligonucleotides (natural or modified) or involve over-expression of such miRNAs from an introduced viral vector. Antagomirs are synthetic oligonucleotide sequences that are designed to be inversely oriented (antisense) to miRNAs that are over-expressed in the disease state and which can form Watson-Crick base pairing with the target miRNA. This can either inactivate a miRNA or result in its degradation. Similar to the miRNA mimics, these therapeutic and research tools can also consist of synthetic or modified nucleic acid sequences or be overexpressed from viral vectors.

Alternative methodologies used in experimental settings include miRNA sponges, which are exogenous DNA repeats of the target sequence and can serve to soak up excess copies of the excess miRNA (Ebert et al., 2007). The miRNA sponges may be produced under the regulation of RNA Polymerase III promoters and can generate high amounts of specific target sequences. Another novel yet promising approach involves target protection. In this application, modified antisense oligonucleotides such as LNA or morpholinos are prepared that will be complementary to a specific sequence in the target gene messenger RNA. These are added to the cells, where they bind to the target sequence, block its down-regulation by the miRNA complex and ensure sufficient expression of the target mRNA (Choi et al., 2007). Enhanced and prolonged miRNA suppression and simultaneous targeting of multiple miRNAs can be achieved by inhibitors carrying clustered hairpins based on the “Tough decoy” (TuD) design which offer the advantage of standardized suppression of families or clusters of miRNAs and can be combined with recombinant adenovirus vectors (Haraguchi et al., 2009; Xie et al., 2012; Bak et al., 2013; Hollensen et al., 2013).

An important difficulty that may be predicted for developing neuronal miRNA therapeutics is delivery, since targeting to the brain involves the significant hurdle of crossing the blood–brain barrier. Nevertheless, therapeutic efficacy of certain approaches such as the use of LNA antagomirs has been demonstrated even in primate models, and certain neuronal miRNA therapeutic approaches are now in preclinical development. These studies cover several creative approaches that have been developed to overcome the delivery problem. Thus, ~20-mer miRNA-size oligonucleotides are indeed unlikely to cross the blood–brain barrier. However, peripheral administration of oligonucleotide controllers of inflammation-regulating miRNAs would change the levels of cytokines, and cytokines can penetrate and affect the brain. Such effects have been demonstrated for miR-132 (Shaked et al., 2009) and miR-212 (Hollander et al., 2010). Other means include direct infection of cerebral neurons with viral vectors that may be adapted for better tropism to neuronal cells (Barbash et al., 2013). Direct introduction of antisense oligonucleotides can alternatively be performed by intracerebroventricular or local stereotactic injection though these would be extremely problematic in pain syndromes. Yet more recent work described the use of rabies virus glycoprotein labeled nanoparticles to enable direct delivery of a miRNA mimic to neuronal cells (Hwang do et al., 2011).

## CONCLUSION

Recently, specific miRNAs have been associated with pathological pain and the deregulation of ion channel expression in sensory neurons in rodent pain models (Zhao et al., 2010; Favereaux et al., 2011; Li et al., 2011). Pain conditions have been suggested to deregulate the expression of miRNAs in pain pathways from primary afferent nociceptors to brain areas associated with emotional components of pain perception (Bai et al., 2007; Aldrich et al., 2009; Imai et al., 2011; Kusuda et al., 2011; Poh et al., 2011; von Schack et al., 2011; Arai et al., 2013; Genda et al., 2013; Sakai and Suzuki, 2013). Unique signatures of miRNAs are associated with altered innate immune signaling and secreted miRNAs are even considered a new form of neuroimmune communication and control immune cell activity as well as neuron function (Peng et al., 2010; Bredy et al., 2011; Chen et al., 2012; Ponomarev et al., 2013). miRNAs act at the neuro-immune interface which controls neuronal plasticity and memory but also are linked to the etiology of anxiety and mood disorders (Bredy et al., 2011; Soreq and Wolf, 2011; O’Connor et al., 2012; Shaltiel et al., 2013). Such deficits in the interaction of immune cells and neurons together with cognitive and emotional alterations in patients with neuropathic or neurogenic pain syndromes are hypothesized to converge on miRNA deregulated mechanisms along the entire neuraxis, and alterations in miRNA expression may account for the variation of susceptibility to certain types of pain or even for the responsiveness to analgesics and opioid tolerance (Parsons et al., 2008). Understanding the role of miRNAs in pain mechanisms is suggested to provide great benefit for clinical diagnostic and therapeutic applications.

## Conflict of Interest Statement

The authors declare that the research was conducted in the absence of any commercial or financial relationships that could be construed as a potential conflict of interest.
